# An ancestry-enriched HNF4A variant and GP2 reveal distinct mechanisms of type 2 diabetes in exome-wide study of 13,674 cases and 41,024 controls

**DOI:** 10.1101/2025.09.24.25336527

**Published:** 2026-05-19

**Authors:** Sam Hodgson, Vi Bui, Siqi Hu, Cyrielle Maroteau, Margherita Bigossi, Alicia Huerta-Chagoya, Trang Nguyen, Adem Y. Dawed, Ryan Koesterer, Maheak Vora, Daniel Stow, Alice Williamson, Alexandra M Blee, Julia Carrasco-Zanini Sanchez, Viswanathan Baskar, Saravanan Jebarani, Benjamin M Jacobs, Georgios Kalantzis, Stuart Rison, Klaudia Walter, Erwan Pennarun, Katherine Taylor, Sarah Hsu, Genes & Health Research Team, MDRF Research Team, Alisa Manning, Miriam Udler, Hilary C Martin, Inês Barroso, Jason Flannick, Matteo Fumagalli, Venkatesan Radha, Rajendra Pradeepa, Claudia Langenberg, Viswanathan Mohan, Ranjit Mohan Anjana, David A van Heel, Josep M. Mercader, Yalda Jamshidi, Sarah Finer, Amit R. Majithia, Moneeza K Siddiqui

## Abstract

Type 2 diabetes (T2D) is a common and complex metabolic condition with significant heterogeneity within and across ancestries ^1–4^ . Compared with individuals of European ancestry (EUR), people of south Asian ancestry (SAS) have two to four-fold higher risk of T2D, develop the disease at younger ages and lower body mass index (BMI), and experience more rapid progression to complications ^5–10^ . Understanding the genetic basis of this is hindered by low representation of south Asians in genetic studies. Here, we perform an exome-wide association study of T2D in 13,674 cases and 41,024 controls from the Genes & Health study of British Pakistani and Bangladeshi individuals. We identify a novel rare variant in *HNF4A* – a canonical monogenic diabetes / MODY gene, in which missense variants would be expected to increase T2D risk. Surprisingly, *HNF4A* Pro437Ser is associated with a halved risk of T2D and reduced risk of diabetes-related complications but increased non-HDL cholesterol. We additionally characterise a T2D risk-increasing variant which is common only in South and East Asian ancestral groups ( *GP2* Val429Met), which is associated with lower BMI and phenotypic and genetic markers of insulin deficiency. We validate our findings through replication in independent multi-ancestry cohorts, *in vitro* functional assays, and integration of proteogenomic analysis. These findings highlight how the study of under-represented populations can identify biological mechanisms associated with disease phenotypes enriched in those populations.

## Full Text Availability

The license terms selected by the author(s) for this preprint version do not permit archiving in PMC. The full text is available from the preprint server.

